# Books Review

**Published:** 1871-07

**Authors:** 


### Books Review.

*The Eye in Health and Disease.* By B. JOY JEFFRIES, A. M., M. D.

The following subjects are presented in language adapted io the undestanding of the general public. The chief object of publication appears to be to furnish the general reader with adequate and reliable information upon the Eye: Anatomy of the Eye. Physiology of the Eye. Old Sight and Spectacles. Near-Sightedness, or Myopia. Long-Sightedness, or Over-Sightedness --Hypermetropia. Astigmatism. Cataract in Children simulating Near-Sightedness. Cataract. Artificial Eyes—How and Why they are Worn. Squinting Eyes--Why and How they must be operated on. An Artificial Pupil--What it is, How and Why the Operation is performed. The Ophthalmoscope--What it is, and how it is used. Injuries and Diseases of the Lids and Eye—Their General Care and Treatment. Type for Testing Vision.

The fact that the articles are adapted to the comprehension of the general reader does not detract from its value as a manual for the student and practitioner. The scientific facts, and practical instructions it contains, should, at least, be thoroughly understood by all practicing physicians.

— —

*Atlantic Monthly.*

We have to acknowledge the regular receipt of the Atlantic Monthly, which is always welcomed by the home circle with great pleasure. This journal may truly be regarded as one of the necessities of all well regulated and intelligent families. It is thus appreciated by all who regularly receive it.

—

*Detection of Criminal Abortion.* By ELY VAN DE WARKER, M. D.

This pamphlet is a guide to the practitioner, containing a table of the differential diagnosis of accidental and spontaneons from instrumental abortion to the the third month, and also a table exhibiting the differences between dysmenorrhœa and instrumental abortion. This pamphlet is a reprint from the Gynæcological Journal of Boston.

—

*Leffel's Illustrated Mechanical News.*

Among the popular and useful journals of its class in the country is Leffel's

Illustrated Mechanical news. Each number contains from eight to twelve illustrations, with a large amount of reading matter pertaining to all branches of mechanical science. It is published in Springfield, Ohio.

---

*Physiological effects of Severe and Protracted Muscular Exercise with special reference to its influence upon the Excretion of Nitrogen.* By AUSTIN FLINT, Jr., M. D.

Under the above title, Dr. Flint, the well known Physiologist, has layed a reprint from the New York Medical Journal, before the medical profession, containing his researches, mainly of the relation of urea to exercise. The observations were taken from Mr. Weston during his extensive walk, and were made at three different periods, of five days each. During the first period, five days before the walk, the average excretion of nitrogen in the urea and fæces amounted to 95.53 parts, for every 100 parts of nitrogen of food. During the second period, five days during the walk, the average excretion of nitrogen in the urea and fæces amounted to 174.81 parts for every 100 parts of nitrogen taken in with the food, while during the third period, five days after the walk, the average excretion of nitrogen in the urea and fæces amounted to 91.93 parts per 100 parts taken with the food.

It will be seen from the above figures, that muscular exercise greatly influences the elimination of nitrogen.

